# N-acetyltransferase 10 regulates alphavirus replication via N4-acetylcytidine (ac4C) modiﬁcation of the lymphocyte antigen six family member E (LY6E) mRNA

**DOI:** 10.1128/jvi.01350-23

**Published:** 2024-01-03

**Authors:** Yamei Dang, Jia Li, Yuchang Li, Yuan Wang, Yajing Zhao, Ningbo Zhao, Wanying Li, Hui Zhang, Chuantao Ye, Hongwei Ma, Liang Zhang, He Liu, Yangchao Dong, Min Yao, Yingfeng Lei, Zhikai Xu, Fanglin Zhang, Wei Ye

**Affiliations:** 1 Department of Microbiology, Airforce Medical University (Fourth Military Medical University), Xi’an, Shaanxi, China; 2 Department of Neurology, Xi’an International Medical Center Hospital, Xi’an, Shaanxi, China; 3 State Key Laboratory of Pathogen and Biosecurity, Beijing Institute of Microbiology and Epidemiology, AMMS, Beijing, China; 4 Department of Oral and Maxillofacial Surgery, State Key Laboratory of Military Stomatology, National Clinical Research Center for Oral Diseases, Shaanxi Key Laboratory of Stomatology, School of Stomatology, Airforce Medical University (Fourth Military Medical University), Xi’an, Shaanxi, China; 5 Key Laboratory of Resources Biology and Biotechnology in Western China, Ministry of Education, College of Life Sciences, Northwest University, Xi’an, Shaanxi, China; 6 Department of Pathogenic Biology, School of Preclinical Medicine, Yan’an University, Yan’an, Shaanxi, China; 7 Department of Infectious Diseases, Tangdu Hospital, Airforce Medical University (Fourth Military Medical University), Xi’an, Shaanxi, China; University of North Carolina at Chapel Hill, Chapel Hill, North Carolina, USA

**Keywords:** N4-acetylcytidine (ac4C), N-acetyltransferase 10 (NAT10), Sindbis virus (SINV), alphavirus, lymphocyte antigen 6 family member E (LY6E)

## Abstract

Epitranscriptomic RNA modifications can regulate the stability of mRNA and affect cellular and viral RNA functions. The N4-acetylcytidine (ac4C) modification in the RNA viral genome was recently found to promote viral replication; however, the mechanism by which RNA acetylation in the host mRNA regulates viral replication remains unclear. To help elucidate this mechanism, the roles of N-acetyltransferase 10 (NAT10) and ac4C during the infection and replication processes of the alphavirus, Sindbis virus (SINV), were investigated. Cellular NAT10 was upregulated, and ac4C modifications were promoted after alphavirus infection, while the loss of NAT10 or inhibition of its N-acetyltransferase activity reduced alphavirus replication. The NAT10 enhanced alphavirus replication as it helped to maintain the stability of lymphocyte antigen six family member E mRNA, which is a multifunctional interferon-stimulated gene that promotes alphavirus replication. The ac4C modification was thus found to have a non-conventional role in the virus life cycle through regulating host mRNA stability instead of viral mRNA, and its inhibition could be a potential target in the development of new alphavirus antivirals.

**IMPORTANCE:**

The role of N4-acetylcytidine (ac4C) modification in host mRNA and virus replication is not yet fully understood. In this study, the role of ac4C in the regulation of Sindbis virus (SINV), a prototype alphavirus infection, was investigated. SINV infection results in increased levels of N-acetyltransferase 10 (NAT10) and increases the ac4C modification level of cellular RNA. The NAT10 was found to positively regulate SINV infection in an N-acetyltransferase activity-dependent manner. Mechanistically, the NAT10 modifies lymphocyte antigen six family member E (LY6E) mRNA—the ac4C modification site within the 3′-untranslated region (UTR) of LY6E mRNA, which is essential for its translation and stability. The findings of this study demonstrate that NAT10 regulated mRNA stability and translation efficiency not only through the 5′-UTR or coding sequence but also via the 3′-UTR region. The ac4C modification of host mRNA stability instead of viral mRNA impacting the viral life cycle was thus identified, indicating that the inhibition of ac4C could be a potential target when developing alphavirus antivirals.

## INTRODUCTION

The post-transcriptional modiﬁcation of nucleic acids within messenger RNAs (mRNAs) has emerged as a significant contributor to RNA metabolism and function regulation (1). These chemical mRNA modifications have been described as epitranscriptome modifications, and they can influence pre-mRNA splicing, localization, transport, stability, and translation. Specifically, epitranscriptome modifications, such as N6-methyladenosine (m6A), N1-methyladenosine (m1A), and 5-methylcytosine (m5C), play important roles in regulating the functions of cellular and viral RNAs, while N4-acetylcytidine (ac4C) modification promotes mRNA stability and translation (2, 3) and is mediated by cellular N-acetyltransferase 10 (NAT10). More specifically, ac4C modifies human immunodeﬁciency virus 1 (HIV-1) and enterovirus 71 (EV71) genomes, which promote viral replication by enhancing the stability of the viral RNA (4, 5).

In addition to direct viral genome modifications, other mechanisms related to RNA modifications contribute to virus replication. For instance, the m6A methyltransferase complex promotes the antiviral activity of type I interferon (IFN) (6), while also regulating the replication of vesicular stomatitis virus, human respiratory syncytial virus, human metapneumovirus, and other negative-sense RNAs via the interferon pathway (7–10), while m6A modifications regulate intestinal immunity and rotavirus infection (11). Furthermore, interferon-stimulated gene 20 electively degrades m6A-modified hepatitis B virus (HBV) transcripts, highlighting the dual role of the host IFN system in combating viral infections (12). Furthermore, HBV induces an increase in the m6A modification of a tumor suppressor transcript, phosphatase and tensin homolog, and thus contributes to the development of hepatocellular carcinoma (13). However, the ac4C modifications in host mRNA instead of viral RNA that regulate virus replication still need to be defined.

Alphaviruses contribute significantly to the global health burden owing to their wide geographic distribution and the resulting disease severity in both humans and other animals. These viruses cause acute polyarthritis and encephalitis in humans and are often transmitted by mosquitos. Specifically, new-world alphaviruses, such as Eastern equine encephalitis virus, Western equine encephalitis virus, and Venezuelan equine encephalitis virus, can cause severe encephalitis and have high mortality rates, making them potential biodefence agents. Furthermore, old-world alphaviruses, including Chikungunya virus (CHIKV), have re-emerged in the Americas, resulting in endemic outbreaks. Elucidating the replication mechanisms of alphaviruses could thus aid in the development of broad-spectrum host-targeting antivirals.

The epitranscriptomic modifications associated with alphavirus genomes are still in need of characterization. The m6A modification has been documented in the CHIKV genome (14). As the prototype *alphavirus*, Sindbis virus (SINV) is an enveloped single-stranded RNA virus that encodes non-structural and structural proteins. The non-structural proteins (NSPs) are cleaved into NSP1–4, and the structural proteins into the capsid, envelope (E)3, E2, and 6K-E1 (15). Moreover, SINV can be readily handled under biological safety level 2 conditions (16), making it an ideal model for studying *alphavirus* replication mechanisms.

In this study, we examined the role of NAT10 and ac4C modifications in regulating genes associated with SINV infection and replication at the mRNA level. The results revealed that SINV infection enhances NAT10 and promotes ac4C modification in host cells and demonstrated that interference with NAT10 significantly reduces SINV infection. Further studies showed that NAT10 promotes SINV replication by relying on its N-acetyltransferase activity. In addition, we found that NAT10 maintains *LY6E* mRNA stability, which enhances SINV replication. Our results reveal a novel mechanism by which NAT10 mediates the regulation of *alphavirus* replication; consequently, NAT10 may be a potential target for the development of new *alphavirus* antivirals.

## MATERIALS AND METHODS

### Cells, viruses, and reagents

A549 (ATCC; CCL-185), Human Microglia Clone 3 (HMC3; CRL-3304, Procell Life Science & Technology Co., Ltd, Wuhan, China), and Huh7 cells were cultured in Dulbecco’s modified Eagle’s medium (DMEM, Sigma-Aldrich, St. Louis, MO, USA) supplemented with 10% fetal bovine serum (FBS, Sigma-Aldrich) in 5% CO_2_ at 37°C. SINV S.A.AR86 strains were stored in our laboratory and propagated in C6/36 cells (ATCC; CRL-1660) and titrated using a plaque formation assay in Vero E6 cells (ATCC; CCL-81).

Rabbit polyclonal antibodies against SINV capsid were generated with synthesized polypeptide-conjugated keyhole limpet hemocyanin immunogen by a commercial company (GenScript, Nanjing, China). Antibodies against NAT10 (cat. no. 13365-1-AP, 1:2,000, ProteinTech, Wuhan, China), LY6E (cat. no. A09496-2, 1:1,000, Boster, Wuhan, China), dsRNA/J2 (cat. no. 10010200, 1:400, Scicons, Szirak, Hungary), β-tubulin (cat. no. D198906, 1:5,000, Sangon Biotech, Shanghai, China), and ac4C (cat. no. ab25251125, 1:500, Abcam, Cambridge, USA) were also used. The secondary antibodies Alexa 488-conjugated goat-anti-mouse, Cy3-conjugated goat anti-rabbit, goat anti-rabbit or mouse IgG (H+L) HRP (Sangon Biotech), and normal rabbit IgG isotype control (Cell Signaling Technology, Danvers, MA, USA) were also used.

Remodelin was obtained from TargetMol (Shanghai, China), while actinomycin D was obtained from MedChemExpress (Monmouth Junction, NJ, USA).

### Plasmid construction

All target genes were cloned into the pCAGGS vector between the *Eco*R I and *Kpn* I sites, in frame with the sequences encoding the GGGGS linker and Myc tag between the *Kpn* I and *Xho* I sites, as previously reported (17), unless otherwise indicated. Clones were constructed using the Hieff Clone Plus One Step Cloning Kit (Yeasen Biotechnology, Shanghai, China), followed by standard bacterial transformation. Site-directed mutagenesis was conducted using overlap PCR with two segments. The K290A and G641E mutants were constructed using overlap PCR and the primers listed in Table 1.

**TABLE 1:** Primer sequences used for molecular cloning

Name	Sequence (5′−3′)
NAT10-F	CATTTTGGCAAAGAATTCAGCCACCATGCATCGGAAAAAGGTGGATAACCGAATCC
NAT10-R	GCCTCCACCCCCGGTACCCTATTTCTTCCGCTTCAGTTTCATATCTTTTTTGTTCTTTG
NAT10-K290A Seg 1-R	TGCTCCCCGTCCTCGAGCAGCTGTG
NAT10-K290A Seg 2-F	GCTGCTCGAGGACGGGGAGCATCTGCAGCCCTGGGATTGGCGATTG
NAT10-G641E Seg 1-R	CTCATAGCCCATCCCTTGATAATCTGGGTG
NAT10-G641E Seg 2-F	GATTATCAAGGGATGGGCTATGAGAGCCGTGCTCTGCAGC
LY6E-F	CATTTTGGCAAAGAATTCGCCACCATGAAGATCTTCTTGCCAGTGC
LY6E-R	GAGCCTCCACCCCCGGTACCGGGGCCAAACCGCAGCAGG

### Short hairpin RNA-mediated gene knockdown

For the knockdown (KD) experiments, the coding sequences for the NAT10-targeting short hairpin RNA (shRNA) were cloned into the pLKO.1-TRC vector (Addgene, #10878). Lentiviruses were obtained by co-transfection of the shRNA with packaging vectors (PsPAX2 and pMD2.G) into 293T cells, according to the Hieff *Trans* Lipofectamine Reagent (Yeasen Biotechnology) protocol. The target sequences were listed as follows: shNAT10 #1: 5′-CGGCCATCTCTCGCATCTATT-3′; shNAT10 #2: 5′-AGGGCCCTCCTTTCCTATAAG-3′; shLY6E: 5′-CCAGAGCTTTCTGTGCAATTT-3′. The virus supernatant was harvested and stored at −80°C. The Huh7 and A549 cells were transduced with the lentivirus containing shNAT10 or shLY6E to yield NAT10 and LY6E stable-knockdown cells, which were then selected with 2 µg/mL puromycin. The transduction efficiency was determined via western blotting and real-time quantitative PCR (qRT-PCR).

### RNA isolation and qRT-PCR

Total RNA was extracted using a Total RNA Kit (Omega Bio-Tek, Georgia, USA) and reverse-transcribed to cDNA using PrimeScript RT Master Mix (Yeasen Biotechnology), according to the manufacturer’s instructions. The target genes were detected via qRT-PCR using SYBR Green Master reagents (Yeasen Biotechnology) on a CFX96 Real-Time system (Bio-Rad, Hercules, CA, USA). Glyceraldehyde-3-phosphate dehydrogenase (*GAPDH*) was used as the internal control for measuring gene expression levels. The relative expression levels of the target genes were evaluated using the 2^−ΔΔCT^ method. The primers used for gene amplification are listed in Table 2.

**TABLE 2:** Primer sequences used for qRT-PCR

Gene name	Sequence (5′−3′)
*NAT10*	Forward: AGTGGTCATCCTCCTACGGAC
	Reverse: TGTACCTGGAATGCACATCCAT
*LY6E*	Forward: CAGCTCGCTGATGTGCTTCT
	Reverse: CAGACACAGTCACGCAGTAGT
*GAPDH*	Forward: ACCCACTCCTCCACCTTTG
	Reverse: ATCTTGTGCTCTTGCTGGG
*SINV*	Forward: CCTTGAAGCGCGTAACATCG
	Reverse: CCGGGCTTCAACTCCTTCTT
*PTPRS*	Forward: CGGGATGAAAACGTGTACGAG
	Reverse: CCCATGTCGATGTTGGGGAA
*NOTCH3*	Forward: TCATGCTGGCTTCTCAGGT
	Reverse: GGTTTCCAGAGAAACCAGT
*TRIM56*	Forward: GCCTGCATACCTACTGCCAAG
	Reverse: GCAGCCCATTGACGAAGAAGT
*RNF135*	Forward: TACTGGGAAGTGGACACTAGGAATT
	Reverse: CTTGACCATGTGCCATGCA
*GAS6*	Forward: GGTAGCTGAGTTTGACTTCCG
	Reverse: GACAGCATCCCTGTTGACCTT

### Western blotting

Total protein was extracted from cells using an immunoprecipitation assay lysis buffer (radio immunoprecipitation assay lysis [RIPA] buffer, Beyotime, Shanghai, China) containing phosphatase and protease inhibitors (MedChemExpress). The protein samples (30 µg) were separated on a 10% SDS-PAGE gel and transferred to a polyvinylidene fluoride membrane (Merck Millipore, Darmstadt, Germany). The membrane was then blocked with solubilized 5% skimmed milk at room temperature (20°C–25°C) for 1 h and incubated with primary antibodies at 4°C overnight. Subsequently, the membranes were incubated with appropriate secondary antibodies at room temperature for 1 h. The membranes were imaged by first combining Liquid A and Liquid B from the enhanced chemiluminescence detection kit (Abbkine Biotechnology, Atlanta, GA, USA) to configure the corresponding volume of the substrate working solution at a 1:1 ratio. The solution was then dripped evenly onto the blotting membrane and photographed using a Tanon 5200 Chemiluminescence Imager (Tanon, Shanghai, China). The relative signal density was quantified using ImageJ software (18).

### Immunofluorescence assay

Cells were seeded on coverslips in 24-well plates at 1 × 10^5^ cells per well. Following adherence, cells were infected with SINV at a multiplicity of infection (MOI) of 1 for 2 h with rocking every 15 min. After adsorption, the inoculum was removed, and the medium was replenished; 24 h post-infection (hpi), the monolayers were fixed with 4% paraformaldehyde for 15 min at room temperature after being washed thrice with Dulbecco’s phosphate-buffered saline (DPBS) and permeabilized with 0.5% (vol/vol) Triton X-100 in DPBS for 10 min. The plates were then blocked with 3% bovine serum albumin (BSA) in DPBS and incubated with dsRNA-specific mouse monoclonal antibody J2 diluted in DPBS supplemented with 3% BSA and NAT10-specific antibody at 4°C overnight (17). The plates were then incubated with Alexa 488-conjugated goat anti-mouse and Cy3-conjugated goat anti-rabbit secondary antibodies at 37°C for 2 h. Cell nuclei were stained with Hoechst 33258 (Yeasen Biotechnology), and the samples were imaged using a Nikon A1R+ confocal microscope.

### ac4C dot blot assay

Dot blots were performed by denaturing 1 µg of the total RNA at 75°C for 5 min, followed by immediate cooling on ice for 1 min. RNA samples were loaded directly onto a Hymond-N^+^ membrane (Merck Millipore, Darmstadt, Germany) and cross-linked at an UV dose of 150 mJ/cm^2^. The membrane was blocked with solubilized 5% skimmed milk for 1 h and incubated with the rabbit monoclonal anti-ac4C antibody (1:500 dilution) at 4°C overnight. The membrane was then washed thrice with 0.1% TBST, incubated with HRP-conjugated anti-rabbit IgG antibody (1:5,000 dilution) for 1 h at room temperature, and developed with Tanon 5200 Chemiluminescence Imaging. Subsequently, the membrane was incubated with 0.2% methylene blue (Sigma-Aldrich) in 0.4 M sodium acetate for 10–15 min and then washed with ddH_2_O. Relative signal intensity was quantified using ImageJ and normalized to the total RNA levels (as measured by methylene blue).

### Plaque formation assay

The level of SINV secreted into the supernatant was determined using a plaque formation assay. Briefly, the supernatants were diluted 10-fold and used to infect confluent monolayers of Vero E6 cells. After adsorption, the medium was discarded, and each well was immersed in 2 mL of maintenance medium: DMEM supplemented with 2% (wt/vol) carboxymethylcellulose and 2% FBS. The maintenance medium was removed after 96 h, and the cells were fixed with 20% formalin in DPBS. Finally, the plaques were stained with 1% crystal violet for 30 min at room temperature, rinsed with ddH_2_O, and then counted using ImageJ software.

### Remodelin assay

Cells (3 × 10^5^) in 12-well plates were infected with SINV for 2 h and treated with 0.5, 1, 5, 10, or 20 µM Remodelin (MedChemExpress). The cell precipitation and culture supernatant were then collected at 24 hpi. The change in the SINV was determined using western blotting and qRT-PCR. The level of SINV secreted into the supernatant was determined using a plaque formation assay.

### Cytotoxicity assay

Huh7 and A549 cells (1.0 × 10^4^) were inoculated in 96-well plates and incubated overnight. The supernatant was then discarded, and the cells were treated with different concentrations of Remodelin. The blank group contained a medium without drugs and was incubated in 5% CO_2_ at 37°C for 24 h. The medium was then removed, and the 10% Cell Counting Kit-8 (CCK8; TargetMol) solution was added to each well and incubated for 4 h in the dark. The absorbance (A) of each well was measured at 450 nm using a BioTek HT synergy microplate reader.

### Viral attachment and internalization assay

Huh7 NAT10-KD and control cells were seeded into 12-well plates at 4 × 10^5^ cells per well. After the cells had adhered, the plates were washed with DPBS and infected with SINV at MOIs of 5 and 10 on ice for 30 min. The inoculant was removed, and the cells were washed five times with DPBS to remove the remaining unbounded virion. RNA was extracted from the cells and attached virus and detected using qRT-PCR for the attachment assay. For the internalization assay, the cells were incubated at 37°C for an additional 2 h after washing. Then, RNA was extracted and detected as described above in RNA isolation and qRT-PCR.

### RNA-seq and data analysis

Control and NAT10-KD cells were infected with SINV (MOI = 5) for 6 h, and the total RNA was isolated and purified using the TRIzol reagent (Invitrogen, Carlsbad, CA, USA) following the manufacturer’s procedure. The RNA concentration and purity were quantified using NanoDrop ND-1000 (NanoDrop, Wilmington, DE, USA), and its integrity was assessed using Bioanalyzer 2100 (Agilent, Santa Clara, CA, USA) with an RIN number >7.0. The data were confirmed by electrophoresis with denaturing agarose gel. Subsequently, 2 × 150 bp paired-end sequencing (PE150) was performed using Illumina Novaseq 6000 (LC-Bio Technologies Co., Ltd., Hangzhou, China) following the manufacturer’s recommended protocol. The *P*-value and fold change (FC) were then calculated for each gene; *P* < 0.05 and logFC≥ 2 represented differentially expressed genes (DEGs). DEGs were then subjected to enrichment analysis of the Gene Ontology (GO) functions and Kyoto Encyclopedia of Genes and Genomes (KEGG) pathways. The data were analyzed, and a heat map, volcano plots, Venn diagrams, and scatter plots were used (https://www.omicstudio.cn/tool).

### acRIP-qPCR and NAT10 RIP-qPCR

RNA immunoprecipitation (IP) was conducted as previously reported (17). Briefly, cells were detached from the plates by scraping, centrifuged at 1,000 *g* for 5 min, collected, and lysed with IP lysis buffer [20 mM Tris (pH 7.5), 150 mM NaCl, and 1% Triton X-100] then supplemented with 100 U/mL RNase inhibitor (New England Biolabs, Ipswich, MA, USA) on ice for 30 min. The samples were stored in 100 µL of lysis buffer at −80°C. The anti-ac4C, anti-NAT10, and normal rabbit IgG isotype control antibodies were then mixed with pretreated protein A/G magnetic beads (Bimake, Houston, TX, USA) and incubated at 4°C for 2 h. Lysis buffer was added, incubated with the beads at 4°C for 2 h, and then washed extensively with IP lysis buffer supplemented with 100 U/mL RNase inhibitor. The RNA was extracted as described in RNA isolation and qRT-PCR. The RNA was analyzed by qRT-PCR, and an equivalent amount of RNA without immunoprecipitation was used as the input control.

### *In vitro* transcription

The wild-type (WT) or ac4C-mutated LY6E transcript was cloned into a plasmid flanked with the 5ʹ T7 promoter and 3ʹ quadruplicate of the S1m aptamer, followed by the self-cleavable HDV ribozyme and T7 terminator. The plasmid was linearized and transcribed with the Ribo RNAmax-T7 kit (RiboBio, Guangzhou, China) according to the manufacturer’s instructions. The MEGA clear kit (Invitrogen) was then used to purify the synthesized RNA, according to the manufacturer’s instructions. Agarose gel electrophoresis was employed to evaluate the integrity and size of the synthetic RNA.

### RNA pull-down assay

Cellular proteins were extracted from the 293T cells using IP lysis buffer supplemented with 100 U/mL RNase inhibitor. The total protein concentration of the extract was measured using a Pierce BCA Protein Assay Kit (Thermo Fisher Scientific, Waltham, MA, USA) according to the manufacturer’s instructions. Meanwhile, 4 µg of each of the RNA probes was denatured at 90°C for 2 min and immediately placed on ice before incubating with pretreated cell lysates under constant mixing for 1 h at 4°C. Subsequently, 40 µL of streptavidin C1 magnetic beads (Invitrogen) was washed and incubated with the cell lysates containing RNA protein complexes at 4°C for 3 h. The beads were washed five times with lysis buffer, mixed with SDS-PAGE sample loading buffer, and analyzed via western blotting.

### RNA decay assay

NAT10-KD or control cells were treated with 5 µg/mL actinomycin D for 0, 1, 2, or 3 h. The total mRNA was then isolated and used for qRT-PCR to quantify the relative abundance of mRNA (relative to 0 h); *GAPDH* was used as the internal control. The half-life of the target mRNA was calculated to analyze the degradation rate. The mRNA half-life was estimated via linear regression analysis.

### Dual-luciferase assay

A dual-luciferase reporter plasmid pmirGLO vector was used to determine the function of the ac4C modification within the 3ʹ-UTR of the LY6E transcripts. The potential ac4C modification sites were predicted on the PACES website (19). The 3′-UTR of the LY6E transcripts (containing one predicted ac4C motif: 3,420–3,434 nt) was cloned downstream of the firefly luciferase; these sites harbored C to T mutations (C–T mut; Fig. 5G). Next, 300 ng of pmirGLO vector containing WT or C–T mut 3′-UTR was transfected into shCon or shNAT10 Huh7 cells in triplicate. The luciferase activity was assessed for 36 h using the Dual-Luciferase Reporter Assay System (Promega, Madison, WI, USA), according to the manufacturer’s instructions.

### Statistical analysis

Statistical analysis was performed using a two-tailed unpaired *t*-test or one-way or two-way analysis of variance (ANOVA) using the GraphPad Prism software (La Jolla, CA, USA). Data are presented as the means ± standard error of the mean (SEM; *n* = 3 unless otherwise indicated).

## RESULTS

### SINV infection induces NAT10 expression and increases the number of ac4C modifications

**Fig 1:**
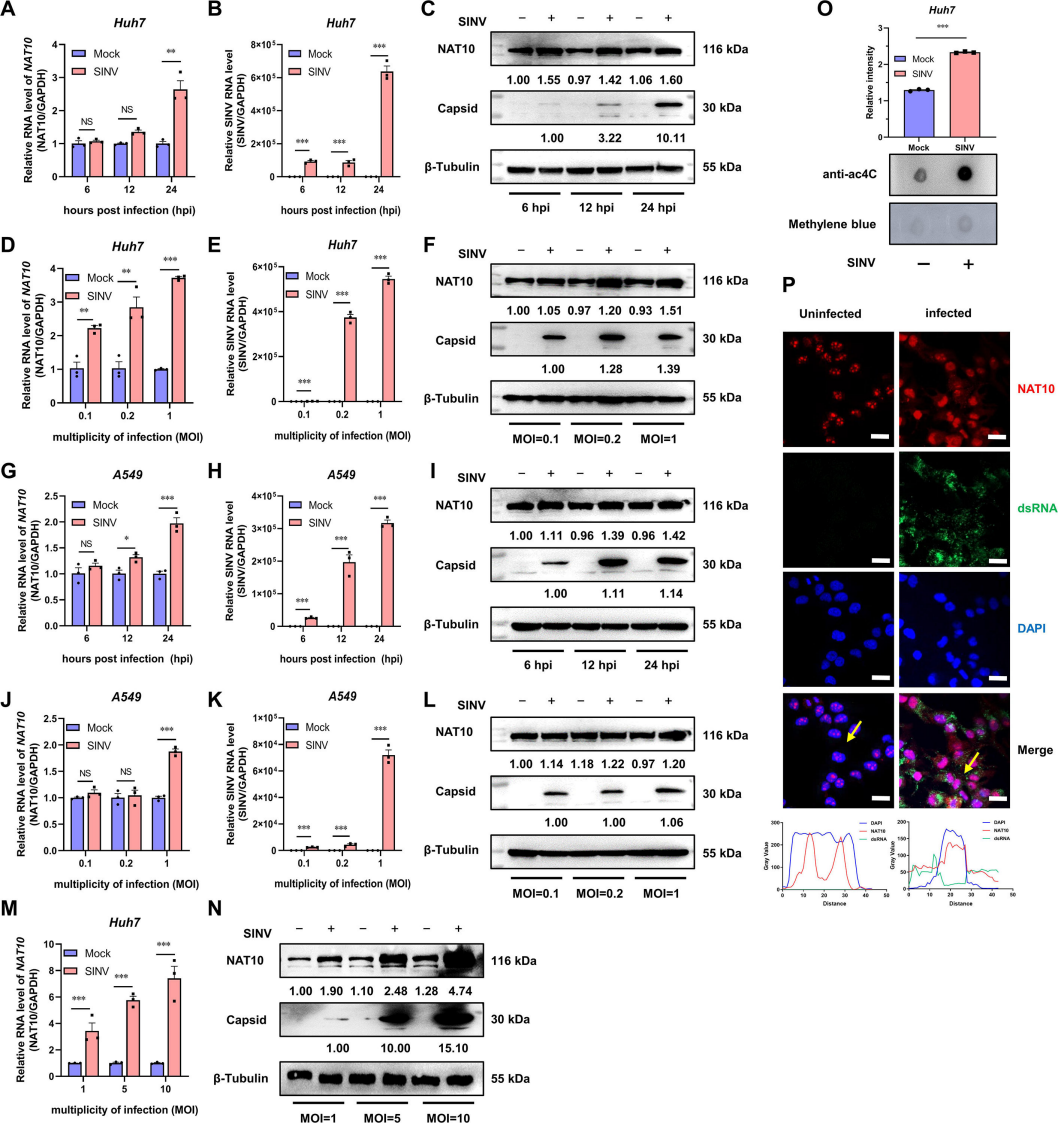
NAT10 expression and ac4C content are increased with SINV infection. (**A**) Huh7 cells were infected with SINV (MOI = 1), and the *NAT10* mRNA was analyzed using qRT-PCR at 6, 12, and 24 hpi. GAPDH was used as the control. (**B**) Huh7 cells were infected with SINV (MOI = 1), and the viral RNA was analyzed using qRT-PCR at 6, 12, and 24 hpi. (**C**) Immunoblot analysis of the NAT10 protein abundance in Huh7 cells infected with SINV (MOI = 1) at 6, 12, and 24 hpi; uninfected cells were used as the control. (**D**) Huh7 cells were infected with SINV (MOI = 0.1, 0.2, and 1). *NAT10* mRNA was analyzed using qRT-PCR at 24 hpi. (**E**) Huh7 cells were infected with SINV (MOI = 0.1, 0.2, and 1), and SINV RNA was analyzed using qRT-PCR at 24 hpi. (**F**) Immunoblot analysis of the NAT10 protein abundance in Huh7 cells at 24 hpi (MOI = 0.1, 0.2, and 1). (**G**) A549 cells were infected with SINV (MOI = 1), and *NAT10* mRNA was analyzed using qRT-PCR at 6, 12, and 24 hpi. (**H**) A549 cells were infected with SINV (MOI = 1), and viral RNA was analyzed using qRT-PCR at 6, 12, and 24 hpi. (**I**) Immunoblot analysis of NAT10 protein abundance in A549 cells infected with SINV (MOI = 1) at 6, 12, and 24 hpi. (**J**) A549 cells were infected with SINV (MOI = 0.1, 0.2, and 1), and *NAT10* mRNA was analyzed using qRT-PCR at 24 hpi. (**K**) A549 cells were infected with SINV (MOI = 0.1, 0.2, and 1), and SINV RNA was analyzed using qRT-PCR at 24 hpi. (**L**) Immunoblot analysis of the NAT10 protein abundance in A549 cells at 24 hpi (MOI = 0.1, 0.2, and 1). (**M**) Huh7 cells were infected with SINV (MOI = 1, 5, and 10). *NAT10* mRNA was analyzed using qRT-PCR at 24 hpi. (**N**) Immunoblot analysis of NAT10 protein abundance in Huh7 cells at 24 hpi (MOI = 1, 5, and 10). (**O**) Total RNA was extracted from SINV-infected and uninfected Huh7 cells (MOI = 1, 24 hpi), blotted with an anti-ac4C antibody (*upper panel*), and stained with 0.2% methylene blue as an internal control (*lower panel*). Relative signal intensity was normalized to total RNA levels (as measured using methylene blue). (**P**) (*Upper panel*) Confocal microscopy of SINV-infected Huh7 cells (MOI = 1, 24 hpi) immunostained for NAT10 (red), dsRNA (green), and nuclei (blue); scale bar = 20 µm. (*Lower panel*) The fluorescence intensity profile of NAT10 (red), dsRNA (green), and nuclei (blue) was measured along the line drawn by ImageJ software. Blots were quantified with ImageJ software and normalized to control levels. Data are presented as the means ± SEM (*n* = 3). ^*^P ≤ 0.05, ^**^P ≤ 0.01, ^***^P ≤ 0.001, and NS, not significant (A, B, D, E, G, H, J, K, and M, two-way ANOVA with Bonferroni post-test; O, unpaired Student’s *t*-tests).

To determine whether SINV infection regulates the NAT10 expression and ac4C modification levels, the NAT10 mRNA levels in SINV-infected cells were analyzed using qRT-PCR and immunoblotting. The NAT10 mRNA level was significantly elevated in the SINV-infected Huh7 cells (Fig. 1A and B), especially 24 hpi. The immunoblotting results confirmed the increase in NAT10 protein abundance in the SINV-infected cells at different time points (Fig. 1C). This was further demonstrated following the infection of Huh7 cells with different SINV MOIs (Fig. 1D and F). To assess the general effect of the SINV on NAT10, experiments were conducted using A549 cells. The NAT10 mRNA and protein expression levels were found to be increased following SINV infection 24 hpi (Fig. 1G through I) and after infection at different MOIs (Fig. 1J through L). In addition, the NAT10 mRNA and protein expression levels were significantly increased after increasing the titer for the SINV infection (Fig. 1M and N). Moreover, the SINV capsid protein level increased in the Huh7 and A549 cells (Fig. 1C, F, I, L, and N). These findings indicate that NAT10 was upregulated after SINV infection, implicating NAT10 in the development of the SINV infection.

As NAT10 impacts the ac4C modification of RNA, we also evaluated the level of ac4C in the total RNA of Huh7 cells infected with SINV. The ac4C level was significantly increased upon SINV infection (Fig. 1O). Additionally, uninfected normal cells expressed NAT10 in the nucleus, especially in the nucleoli, while in the SINV-infected cells, the NAT10 distribution was altered, as it appeared in the nucleus and cytosol (Fig. 1P). Moreover, the cytoplasm and nucleus ratio of the NAT10 increased, suggesting that SINV infection altered the subcellular localization of NAT10. Collectively, these findings demonstrate that SINV infections may enhance NAT10 expression and upregulate the ac4C modification of RNA.

### NAT10 enhances SINV infection

**Fig 2:**
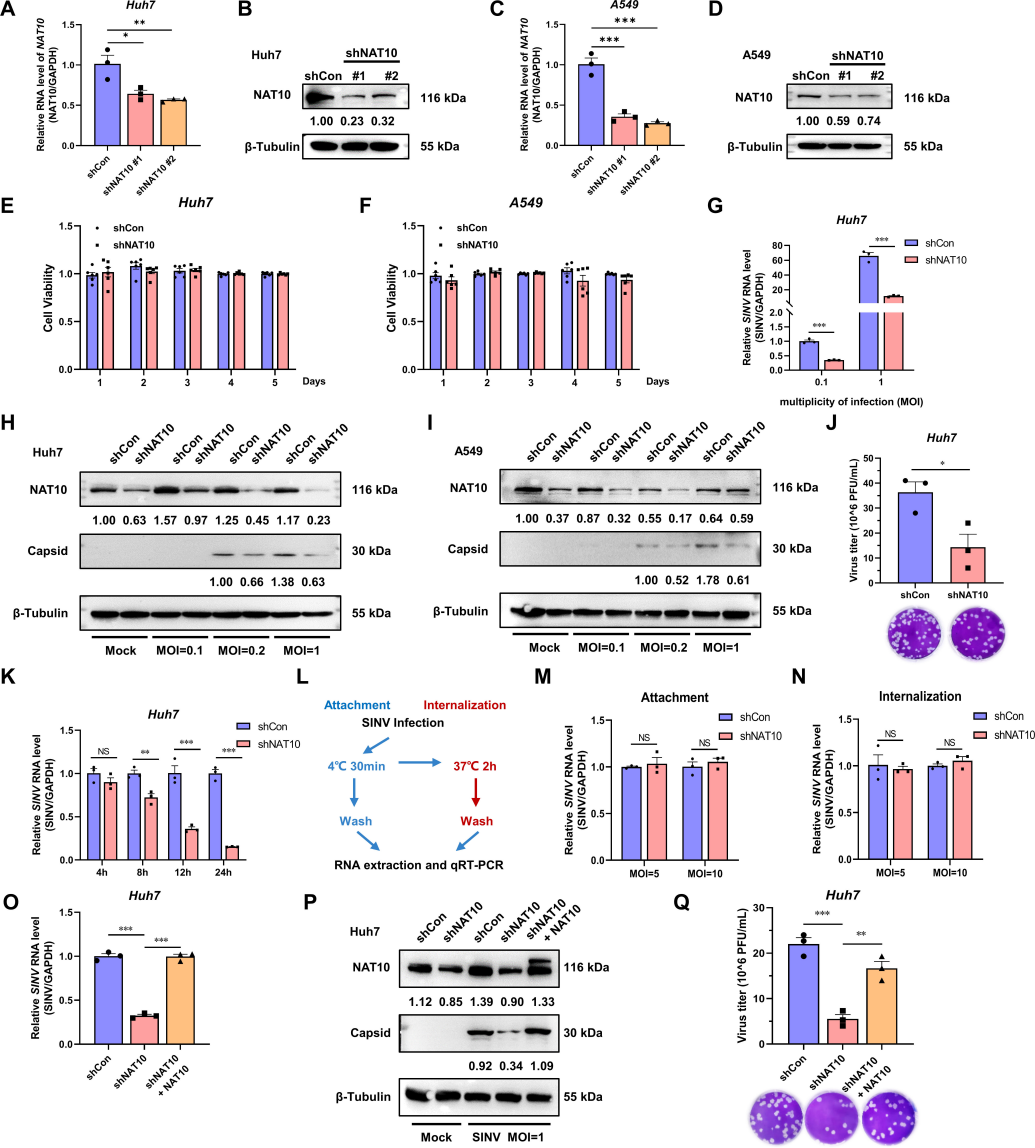
NAT10 is required for SINV replication. (**A**) qRT-PCR analysis of *NAT10* mRNA expression in NAT10 stable-KD Huh7 cells. (**B**) Immunoblot analysis of NAT10-KD Huh7 cells. shNAT10 #2 was used in the subsequent experiments. (**C**) qRT-PCR analysis of *NAT10* mRNA expression in NAT10-KD A549 cells. (**D**) Immunoblot analysis of NAT10-KD A549 cells. shNAT10 #2 was used in the subsequent experiments. (**E, F**) NAT10-KD Huh7 (**E**) cells were evaluated with the A549 (**F**) and control cells using the CCK8 assay for a period of 5 days, during which the 10% CCK8 solution was added at the same time each day, and the absorbance values of each well were detected after 4 h of incubation in the dark (*n* = 6). (**G**) qRT-PCR analysis of the SINV RNA expression levels in the NAT10-KD Huh7 cells at 24 hpi (MOI = 0.1 and 1). (**H, I**) Immunoblotting of the SINV capsid protein abundance in the control and NAT10-KD Huh7 (**H**) and A549 (**I**) cells at 24 hpi (MOI = 0.1, 0.2, and 1). (**J**) SINV infectious virion abundance in Vero E6 cells infected with a 10-fold dilution of NAT10-KD Huh7 cell supernatant (MOI = 1); counted following 1% crystal violet staining after 96 h. (**K**) Huh7 NAT10-KD and control cells were infected with SINV (MOI = 5), and the SINV RNA was assessed using qRT-PCR at 4, 8, 12, and 24 hpi. (**L**) Flowchart showing the viral attachment and internalization assay. (**M**) Attachment assay. SINV RNA was analyzed using qRT-PCR in Huh7 NAT10-KD and control cells infected with SINV (MOI = 5 and 10) for 30 min at 4°C. (**N**) Internalization assay. SINV RNA was analyzed using qRT-PCR in Huh7 NAT10-KD and control cells infected with SINV (MOI = 5 and 10) for 2 hpi at 37°C. (**O, P**) NAT10-KD Huh7 cells exogenously transfected with Myc-tagged NAT10-expressing plasmid and infected with SINV (MOI = 1), qRT-PCR analysis of the SINV RNA expression (**O**); immunoblot analysis of the SINV capsid protein level (**P**) was conducted at 24 hpi. (**Q**) SINV infectious virions in the culture supernatant determined via a plaque formation assay as described for panel (**P**). Blots were quantified with ImageJ software and normalized to control levels. Data are presented as the means ± SEM (*n* = 3). ^*^P ≤ 0.05, ^**^P ≤ 0.01, ^***^P ≤ 0.001, and NS, not significant (A, C, O, and Q, one-way ANOVA with Tukey’s multiple comparisons test; G, K, M, and N, two-way ANOVA with Bonferroni post-test; J, unpaired Student’s *t*-tests).

As SINV regulates the NAT10 level during infection, the role of NAT10 in the SINV infection was investigated. To this end, we generated stable NAT10-KD Huh7 and A549 cell lines using shRNAs targeting the coding sequences of NAT10 (shNAT10). Furthermore, the knockdown was validated using qRT-PCR and immunoblotting (Fig. 2A through D). Importantly, the NAT10 knockdown did not affect Huh7 and A549 cell viability (Fig. 2E and F). Under different MOIs, the SINV RNA level was significantly decreased in the NAT10-KD Huh7 cells (Fig. 2G). Consequently, the SINV capsid protein levels were also significantly reduced in the NAT10-KD Huh7 and A549 cells with an MOI = 1, compared with the control group (Fig. 2H and I). The abundance of infectious SINV virions in the culture supernatant of the NAT10-KD Huh7 cells was significantly downregulated (Fig. 2J).

To understand which stage of the life cycle during the SINV infection the NAT10 plays a role, the Huh7 NAT10-KD and control cells with an SINV at MOI = 5 were used to assess the levels of SINV RNA at different time points post-infection. The SINV RNA in the NAT10-KD cells was significantly reduced at 12 and 24 hpi, suggesting that NAT10 plays a role in the later stages of viral infection (Fig. 2K). In addition, we investigated whether NAT10 affects viral attachment and internalization (Fig. 2L). There were no differences in the SINV RNA between the control and NAT10-KD cells in the viral attachment assay (Fig. 2M), and the viral internalization assay showed similar results (Fig. 2N), suggesting that NAT10 is not required for viral entry. Subsequently, the NAT10 was exogenously expressed in NAT10-KD Huh7 cells, and this resulted in the restoration of the SINV RNA and capsid protein expression levels (Fig. 2O and P), as well as the SINV titer in the culture supernatant (Fig. 2Q). These findings suggest that NAT10 is required for efficient SINV replication.

### N-Acetyltransferase activity of NAT10 is required for SINV infection

**Fig 3:**
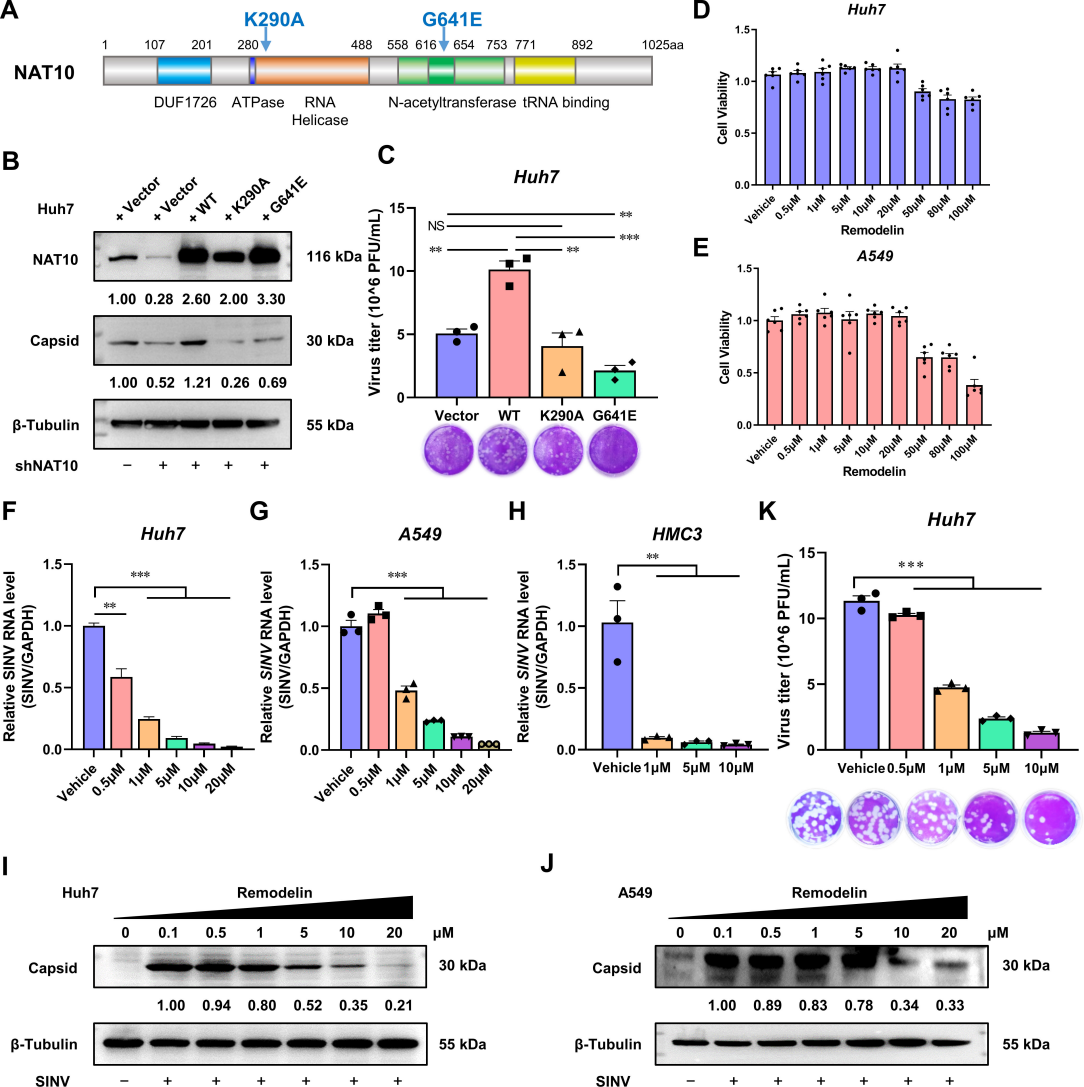
N-Acetyltransferase activity is required for NAT10 to support SINV infection. (**A**) Schematic diagram showing the RNA helicase and N-acetyltransferase domains of NAT10. (**B**) Immunoblotting of the control or NAT10-KD Huh7 cells transfected with plasmids expressing the WT NAT10, K290A mutant, or G641E mutant and infected with SINV for 24 h (MOI = 1). (**C**) Plaque formation assay of the SINV infectious virions in the culture supernatant of panel B. (**D, E**) Viability of Huh7 (**D**) and A549 (**E**) cells at 24 hpi with the Remodelin incubation at different doses, detected using the CCK8 assay. (**F**) qRT-PCR analysis of the SINV RNA expression levels in Huh7 cells infected with SINV and treated with different concentrations of Remodelin (MOI = 1). (**G**) qRT-PCR analysis of SINV RNA expression levels in A549 cells infected with SINV and treated with different concentrations of Remodelin (MOI = 1). (**H**) qRT-PCR analysis of the SINV RNA expression levels in HMC3 cells infected with SINV and treated with different concentrations of Remodelin (MOI = 1). (**I**) Immunoblotting of the SINV capsid protein in Huh7 cells infected with SINV for 24 h and treated with different concentrations of Remodelin (MOI = 1). (**J**) Immunoblotting of the SINV capsid protein in A549 cells infected with SINV for 24 h and treated with different concentrations of Remodelin (MOI = 1). (**K**) Plaque formation assay using the SINV infectious virions in the culture supernatant from the Huh7 cells infected with SINV and treated with different concentrations of Remodelin (MOI = 1). Blots were quantified with ImageJ software and normalized to control levels. Data are presented as the means ± SEM (*n* = 3). ^**^P ≤ 0.01, ^***^P ≤ 0.001, and NS, not significant (C, F, G, H, and K, one-way ANOVA with Tukey’s multiple comparisons test).

NAT10 is the only acetyltransferase that has been identified for RNA, to date, and the RNA helicase and N-acetyltransferase domains are pivotal for catalyzing the properties of NAT10 (Fig. 3A) (2). As NAT10 regulates SINV replication, we hypothesized that these two domains would also regulate SINV infection. To investigate this, an NAT10 K290A mutant, lacking RNA helicase activity, and a G641E mutant, lacking N-acetyltransferase activity, were generated (Fig. 3A) (20). The overexpression of WT NAT10 enhanced SINV infection in the NAT10-KD Huh7 cells; however, ectopic K290A and G641E mutants did not rescue the capsid expression or virus replication levels of the SINV (Fig. 3B and C). Moreover, the expression of G641E mutants reduced the SINV virion level in the supernatants (Fig. 3C); we speculated that this may also be due to a dominant negative effect of the mutant (20, 21). Although the mutated NAT10 was still bound to the substrate, it was not catalytically active. Therefore, it may have interfered with the function of the WT NAT10, resulting in the mutant being unable to rescue the NAT10-promoted viral replication process. Thus, the RNA helicase and N-acetyltransferase domains of the NAT10 are required for efficient SINV replication.

To confirm the importance of N-acetyltransferase, cells were treated with Remodelin—a specific NAT10 N-acetyltransferase inhibitor (20). Upon treatment with 20 µM of Remodelin, which did not affect cell viability (Fig. 3D and E), a dose-dependent decrease in SINV RNA levels was observed in both the Huh7 and A549 cells when compared to that in the control group (Fig. 3F and G). Similar results were observed in HMC3 cells (Fig. 3H). The capsid protein level and viral titer in the culture supernatant were also reduced in the Remodelin-treated group (Fig. 3I and K). These findings highlight the vital role of the N-acetyltransferase activity in relation to NAT10 during an SINV infection.

### Identification of NAT10 targets using RNA sequencing

**Fig 4:**
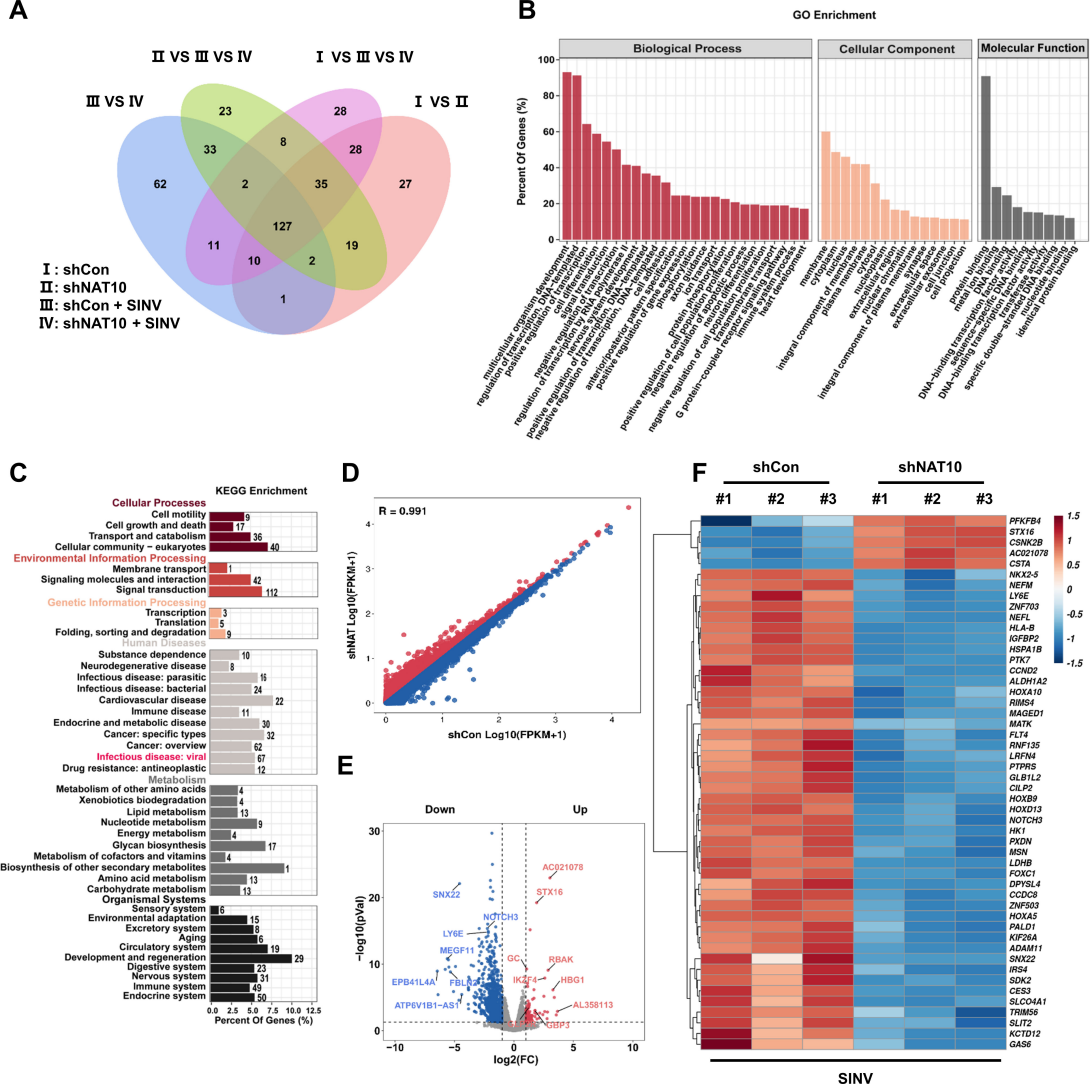
Identification of the NAT10 targets using RNA sequencing. (**A**) Venn diagram showing four Huh7 cell comparison groups with DEGs. (**B**) Functional annotation and pathway enrichment analysis results of predicted downstream target genes of NAT10 are shown. All DEGs were mapped to GO terms in the Gene Ontology database. Gene numbers were calculated for each term, and significantly enriched GO terms for the DEGs compared to those in the background genome were defined using the hypergeometric test. GO terms with a *P* < 0.05 were defined as significantly enriched GO terms in the DEGs. The horizontal axis represents the different GO functional categories, and the vertical axis represents the number of genes within that category as a percentage of the total number of genes for the annotation. (**C**) KEGG enrichment analysis revealed that the DEGs are primarily enriched in infectious diseases. The horizontal coordinate is the percentage of differential genes annotated to the pathway for all differential genes with annotations; the vertical coordinate is the name of the KEGG pathway enriched in differential genes. The bar graphs are colored separately to show the classification of the KEGG pathway. (**D**) Scatter plots of the differential mRNA expression determined from the RNAseq data. Red dots denote upregulated genes, and blue dots denote downregulated genes. (**E**) Volcano plot showing genes with upregulated (red) and downregulated (blue) expression in NAT10-KD cells infected with SINV. Log2 (FC) is the horizontal coordinate, representing the fold change in differential expression of genes across samples; −log10 (*P* value) is the vertical coordinate, representing the significance of the change in expression of the DEGs. (**F**) Heat map showing differential expression clusters for the top 50 genes in NAT10-KD Huh7 cells infected with SINV. The horizontal coordinate is the sample, and the vertical coordinate is the screened DEGs. The change in color from blue to white to red indicates expression from low to high. Red and blue indicate genes with high and low expressions, respectively.

As the SINV infection upregulates the expression of NAT10 and the ac4C modification level in the total RNA, we explored the potential ac4C modification sites in the SINV genome. To this end, AcRIP-seq was adopted; however, no clear reads could be mapped onto the SINV genome. We then assessed whether the NAT10 regulated downstream mRNA to manipulate SINV replication. RNA-seq was performed on NAT10-KD or normal Huh7 cells infected with SINV. A total of 127 DEGs were detected between the NAT10-KD cells and the control group (Fig. 4A). Among them, 97 genes were downregulated in NAT10-KD cells, while 30 were upregulated after SINV infection, indicating that most were downregulated after NAT10 depletion and that the NAT10 knockdown enhanced mRNA transcript reduction after viral infection.

GO enrichment analysis showed that the downregulated DEGs were associated with cell adhesion, transcription factor activity, transmembrane transport, cell junctions, and various other biological processes (Fig. 4B). Meanwhile, KEGG pathway analysis revealed that these DEGs were enriched in biological processes related to infectious diseases and cellular amino acid metabolic processes (Fig. 4C). Volcano and scatter plots also showed significant changes in mRNA abundance in the presence of NAT10 depletion (Fig. 4D and E). To identify target genes that may regulate SINV infection, the top 50 DEGs were clustered (Fig. 4F). Six genes were closely associated with viral infection, namely, *LY6E* (22), receptor-type tyrosine-protein phosphatase S (*PTPRS*) (23), neurogenic locus notch homolog protein 3 (*NOTCH3*) (24), tripartite motif containing 56 (*TRIM56*) (25), ring finger protein 135 (*RNF135*) (26), and growth arrest-specific 6 (*GAS6*; Fig. 4F) (27, 28). These results indicate that NAT10 could modulate the expression of these genes to regulate viral infection.

### LY6E mRNA is a tar**get transcript of NAT10**

**Fig 5:**
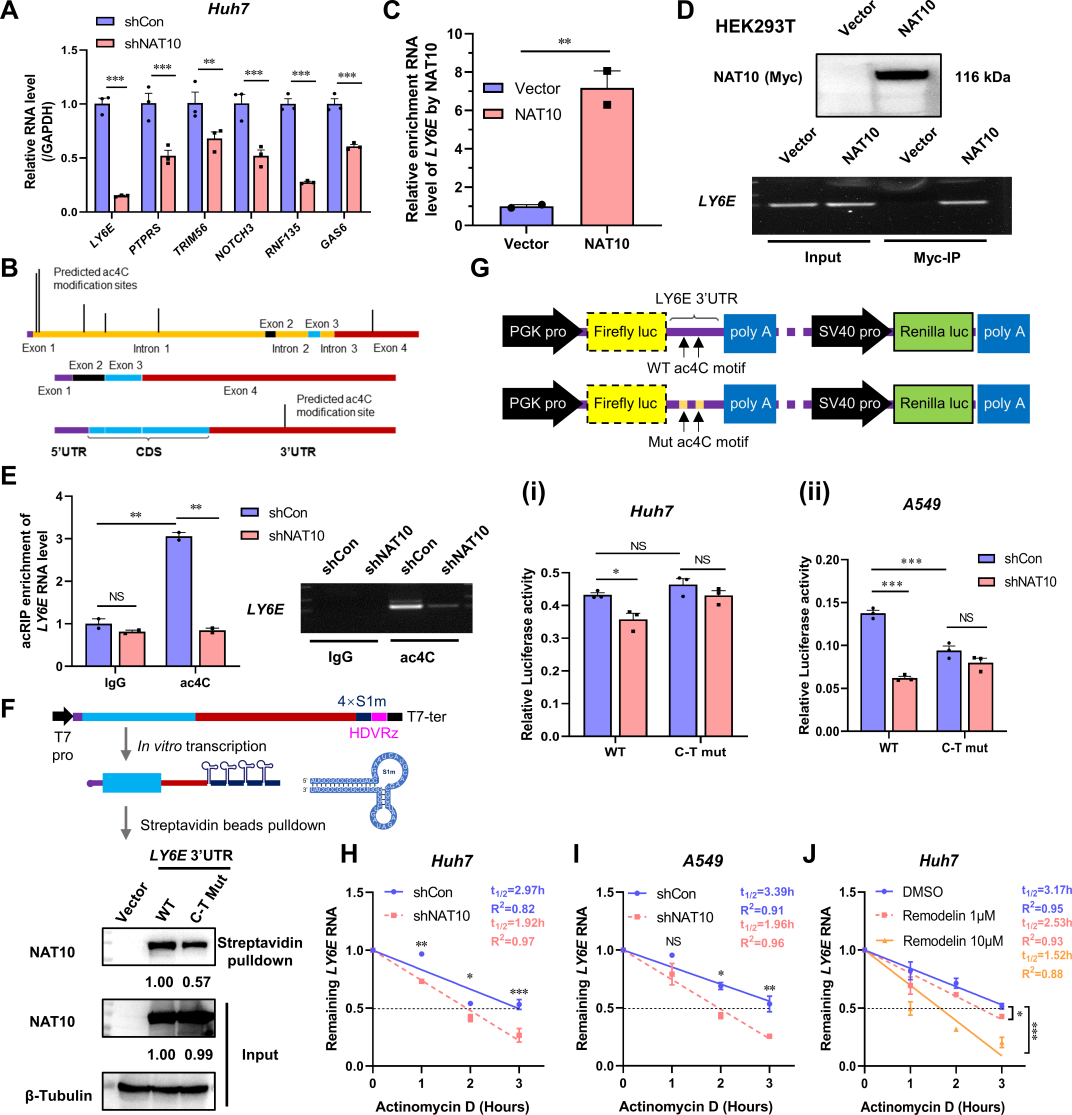
NAT10 targets LY6E, which mediates ac4C modifications during SINV infection. (**A**) Validation of candidate genes from the RNAseq data using qRT-PCR in NAT10-KD cells infected with SINV. (**B**) Predicted ac4C modification sites for the *LY6E* pre-mRNA and mature mRNA. (**C**) IP of 293T cells transfected with the NAT10-Myc plasmid and anti-Myc antibody; enriched *LY6E* mRNA was analyzed using qRT-PCR; the interactions between the NAT10 and *LY6E* mRNA were also analyzed. (**D**) (*Upper panel*) Immunoblot of the NAT10 immunoprecipitate in panel C. (*Lower panel*) Agarose gel electrophoresis images of the *LY6E* amplified using qRT-PCR in panel C. (**E**) After incubating with the anti-ac4C antibody and normal rabbit IgG mixed with protein A/G beads at 4°C for 2 h, respectively, incubation was continued with the NAT10-KD Huh7 cell lysate for 2 h. The bound ac4C-modified RNA was eluted and analyzed using qRT-PCR. (*Left panel*) The ac4C-modified RNA was also analyzed using qRT-PCR. (*Right panel*) Agarose gel electrophoresis images of the *LY6E* amplified using qRT-PCR. Equal amounts of RNA fragments not subjected to immunoprecipitation were used as the input controls. (**F**) (*Upper panel*) Schematic of the 4xS1m aptamer. (*Lower panel*) WT or ac4C site mutated (C–T mut) *LY6E* mRNA tagged with 4xS1m aptamer was incubated with cell lysates overexpressing NAT10 and separated via streptavidin-conjugated beads. NAT10 in the cell lysate was pulled down, and the *LY6E* mRNA was detected using an immunoblot. Cells transfected with vectors were used as negative controls. (**G**) (*Upper panel*) Schematic diagram of the dual-luciferase reporter plasmid pmirGLO. (*Lower panel*) Luciferase activity in the NAT10-KD Huh7 (**I**) or A549 (ii) cells transfected with pmirGLO with the WT or ac4C-modifier-site-mutated (C–T mut) the 3′-UTR of the *LY6E* mRNA. Firefly luciferase activity was normalized to Renilla luciferase activity. (**H, I**) Stability of *LY6E* mRNA in NAT10-KD Huh7 (**H**) and A549 (**I**) cells after treatment with actinomycin D (5 µg/mL) was analyzed using qRT-PCR at different time points. (**J**) *Ly6E* mRNA levels were analyzed in Huh7 cells using qRT-PCR at different time points after 24 h of Remodelin treatment with actinomycin D. Blots were quantified with ImageJ software and normalized to control levels. Data are presented as the means ± SEM (*n* = 3). ^*^P ≤ 0.05, ^**^P ≤ 0.01, ^***^P ≤ 0.001, and NS, not significant (A, E, G, H, I, and J, two-way ANOVA with Bonferroni post-test; C, unpaired Student’s *t*-tests).

The expression levels of *LY6E, TRIM56, PTPRS, GAS6, RNF135*, and *NOTCH3* in the NAT10-KD Huh7 cells were all significantly reduced (Fig. 5). Among these, *LY6E* is a unique interferon-stimulated gene (ISG), also known as a retinoic acid-inducible gene or stem cell antigen 2, that regulates membrane-dependent processes, such as endocytic transport and signaling. It also promotes viral infections, including HIV-1, West Nile, dengue, and Zika (29). Notably, the *LY6E* mRNA level was significantly reduced in the NAT10-KD Huh7 cells (Fig. 5A).

The potential ac4C modification sites in the pre-mRNA and mRNA for LY6E were predicted using the PACES website (19). Several sites were predicated above the threshold score (0.1570) in the first intron of the pre-mRNA, with one possible ac4C site located in the 3ʹ-UTR of the mature *LY6E* mRNA (Fig. 5B, Table 3). Further analysis revealed a conserved ac4C motif within the 3′-UTR of the *LY6E* mRNA, suggesting that NAT10 could directly regulate *LY6E* expression.

**TABLE 3:** Predicted ac4C modification sites in the *LY6E* mRNA determined using PACES^*a*^

ID	Sequence (5′−3′)	Start	End	Score thresholds: 0.1570
1	AGAGCGCGCGAGGTTCGGGGAGCTCGGCCAGGCTGCTGGTACCTGCGTCCGC CCGGCGGTGAGTCCGCGGGCCCCCGGCCGGGACGCCCCCGCCACCTGCGCG CACGCGCTCAGACCCGGCGGCCTCGGCTGCGGTGCACGCGGCCCCGGCTCA GCCACGCGCGGCGGAAGGCGCCCTGCGGGGGCCGGGGCGGGAGAGGGTGG GAGAGAGCAAGAGGGGCGCACGGGGAGGCGCAGGAGAGGGGCTGGGGCTGC CGGTGGGTTGGTCCCCAGAGAGCTGAGCTTCTCCTCCCATCCCCCGC	88	102	0.4991
2	AGAGCGCGCGAGGTTCGGGGAGCTCGGCCAGGCTGCTGGTACCTGCGT CCGCCCGGCGGTGAGTCCGCGGGCCCCCGGCCGGGACGCCCCCGCCA CCTGCGCGCACGCGCTCAGACCCGGCGGCCTCGGCTGCGGTGCACGCG GCCCCGGCTCAGCCACGCGCGGCGGAAGGCGCCCTGCGGGGGCCGGG GCGGGAGAGGGTGGGAGAGAGCAAGAGGGGCGCACGGGGAGGCGCA GGAGAGGGGCTGGGGCTGCCGGTGGGTTGGTCCCCAGAGAGCTGAGC TTCTCCTCCCATCCCCCGCAGGCCTCCCCGAATGTTTCCAAAGATCTGGGGC	121	135	0.5323
3	GCCGCCAGGAGCCTCCCGGCCGCCCCAGGGCTGCGCAGCCACTGG AGCCCATCACCCAACTCCAGACGCTCCTGGCTCCTCTACGTGGGCCGG GAGGGACAGCCTTGAGGACTAGGGGAGGGGGCACGGGACCTTGCAGA GCTCCTGGCCGGAAAGGGAGGATTGACCGCCCCCGGCATATCACCCCG GAGCACTGGAACCCGCCCCCGCTTCTTGTTTTGGCACTGGTGGTGCTTG CGGTGAGGTCCAAGGAGCCCAGCCTCCCTGAGTGGACCGCCGGGCCC TCCCCGTTCCGGGACACAGGAGAGGCTCCCGCCCCTTGCTGGCTGG GCAGCCCCTAGATACCTGGCTCCCAGGGGCCAGCTTCCCTGAGCCTGGG GATGAGCCATGAGCCTGCAGCCTGGGCCCCAGGGG	553	567	0.2356
4	GTTTTGGCACTGGTGGTGCTTGCGGTGAGGTCCAA GGAGCCCAGCCTCCCTGAGTGGACCGCCGGGCCCC TCCCCGTTCCGGGACACAGGAGAGGCTCCCGCCCCTT GCTGGCTGGGCAGCCCCTAGATACCTGGCTCCCAGGG GCCAGCTTCCCTGAGCCTGGGGATGAGCCATGAGCCTG CAGCCTGGGCCCCAGGGGCGCCCCCCACGGCCTGCCGG CTGGCTCCCTCCGGGCTCCATGGCCCACCCGGCCTTCCTAA TTGCCTTCGGCTCCCACCGGTGCCTTAGCTCAGCCTGGTGGC CCAGCGGGTTGGGTGCCACCCAGTGAGCAGGTGGCGGCACC AGCTGGACCTGTTTGTGGCCCTGTATGCTAGGACTTCCTCAGA GACAGCTCAGGGACCCCCCCACACCAGA	768	782	0.1619
5	CACTCCCAACCCCAGGATGTCAGCCCAGGTCTCACTGT CCTGGCCTGCTGCTCTCCCCTTAGGGCCTCTGCGGGCCC CTCTCCAGATCTGTTCTCTGAGGCATCCTCCTTACCCCCAGT GCCCAGCACTAGCTCCCCAGGCCCGGGATGTCCCTCCCACTCCT CTGCCCACGGCTGTCCCTGACAGAAGGCAGCAGCCTCCCCCTCCA ACACCATGCACTCACAAAACAGAGAATCACGACCCCAGCTGGGTGTGTTC CAGATTCTTTCTCCAGCAGTGCAGAGGGTCCTGTGCAGAGGCCGAGGAG CAGTACAGCGACCCATCTGGCCCTTTCCTGCTGGTGGGACCAGTGGCAC GCAGCCTTGTCTCTCGGAGCCCATTTCCCAGCCACAGAATGGGGAGTAGCAGATACTGA	1,303	1,317	0.2012
6^*b*^	CTGCTGAGCCTGCTGCCGGCCCTGCTGCGGTTTGGCCCCTGACCGC CCAGACCCTGTCCCCCGATCCCCCAGCTCAGGAAGGAAAGCCCAGCC CTTTCTGGATCCCACAGTGTATGGGAGCCCCTGACTCCTCACGTGCCT GATCTGTGCCCTTGGTCCCAGGTCAGGCCCACCCCCTGCACCTCCACC TGCCCCAGCCCCTGCCTCTGCCCCAAGTGGGGCCAGCTGCCCTCACT TCTGGGGTGGATGATGTGACCTTCCTTGGGGGACCGCGGAAGGGACG AGGGTTCCCTGGAGTCTTACGGTCCAACATCAGGACCAAGTCCCATGG ACATGCTGACAGGGTCCCCAGGGAGACCGTGTCAGTAGGGATGTGTG CCTGGCTGTGTACGTGGGTGTGCAGTGCACGTGAGAG	3,420	3,434	0.1924

^*a*^ Predicted potential ac4C modification sequences were underlined.

^*b*^ Located within the 3′-UTR of the mature *LY6E* mRNA.

To further validate this hypothesis, Myc-tagged NAT10 was transfected into HEK-293T cells and an RNA immunoprecipitation assay followed by qRT-PCR. LY6E transcript levels were increased, indicating that NAT10 was directly bound to the *LY6E* mRNA (Fig. 5C and D). Moreover, in the NAT10-KD or control cells, the IgG isotype control did not precipitate *LY6E* transcripts; however, the ac4C antibody precipitated a significantly higher level of *LY6E* mRNA (Fig. 5E). Additionally, the WT or ac4C-site-mutated (C–T mut) 3ʹ-UTR of the *LY6E* mRNA was cloned into an S1m aptamer vector (Table 4), and *in vitro* transcription was conducted (7). The 4×S1m aptamer is an RNA fragment capable of binding streptavidin tetramers (30). In the WT 3′-UTR of the *LY6E* mRNA, NAT10 levels were downregulated compared with those in the C–T mut 3′-UTR (Fig. 5F), suggesting that the *LY6E* mRNA was ac4C-modified and regulated by NAT10.

**TABLE 4:** Mutations in the predicted ac4C modification sites of the *LY6E* mRNA^*a*^^,^^*b*^

ID	Sequence (5′−3′)	Start	End	Score thresholds: 0.1570
1	CCCCGCCACCTGCGC ttttGttAttTGtGt	88	102	0.4991
2	CGGCCTCGGCTGCGG tGGttTtGGtTGtGG	121	135	0.5323
3	CCGCCCCCGCTTCTT ttGtttttGtTTtTT	553	567	0.2356
4	CGCCCCCCACGGCCT ttGtttttGtTTtTT	768	782	0.1619
5	CCCCTCCAACACCAT ttttTttAAtAttAT	1303	1317	0.2012
6^*b*^	CTGCCTCTGCCCCAA tTGttTtTGttttAA	3420	3434	0.1924

^*a*^ Underlined potential ac4C modification sequences (upper) were mutated to eliminate C (lower).

^*b*^ Located within the 3′-UTR of the mature *LY6E* mRNA.

As ac4C in the CDS and the 5′-UTR affects mRNA stability, it was suggested that the ac4C site within the 3′-UTR of the *LY6E* mRNA may have a similar effect. To test this hypothesis, luciferase reporter assays were conducted. Compared with that in the control cells, the luciferase activity of the WT *LY6E* 3′-UTR constructs was significantly reduced in the NAT10-KD cells (Fig. 5G). However, in the NAT10-KD Huh7 cells, the luciferase activity was not altered when a reporter construct harboring an ac4C site mutation (C–T mut) was introduced into the 3′-UTR of the *LY6E* mRNA (Fig. 5G).

The half-lives of the *LY6E* mRNA NAT10-KD and the control cells treated with actinomycin D to prevent new transcript mRNA synthesis were then evaluated. That of the *LY6E* mRNA was significantly decreased after the NAT10 knockdown (Fig. 5H and I). Enhanced degradation of the *LY6E* mRNA was further observed using the NAT10 inhibitor Remodelin in Huh7 cells (3 hpi), indicating a loss of *LY6E* mRNA stability with NAT10 inhibition (Fig. 5J). Thus, the potential ac4C site in the 3′-UTR may contribute to the *LY6E* mRNA stability and be regulated by NAT10.

### NAT10 enhances SINV infection via the LY6E

**Fig 6:**
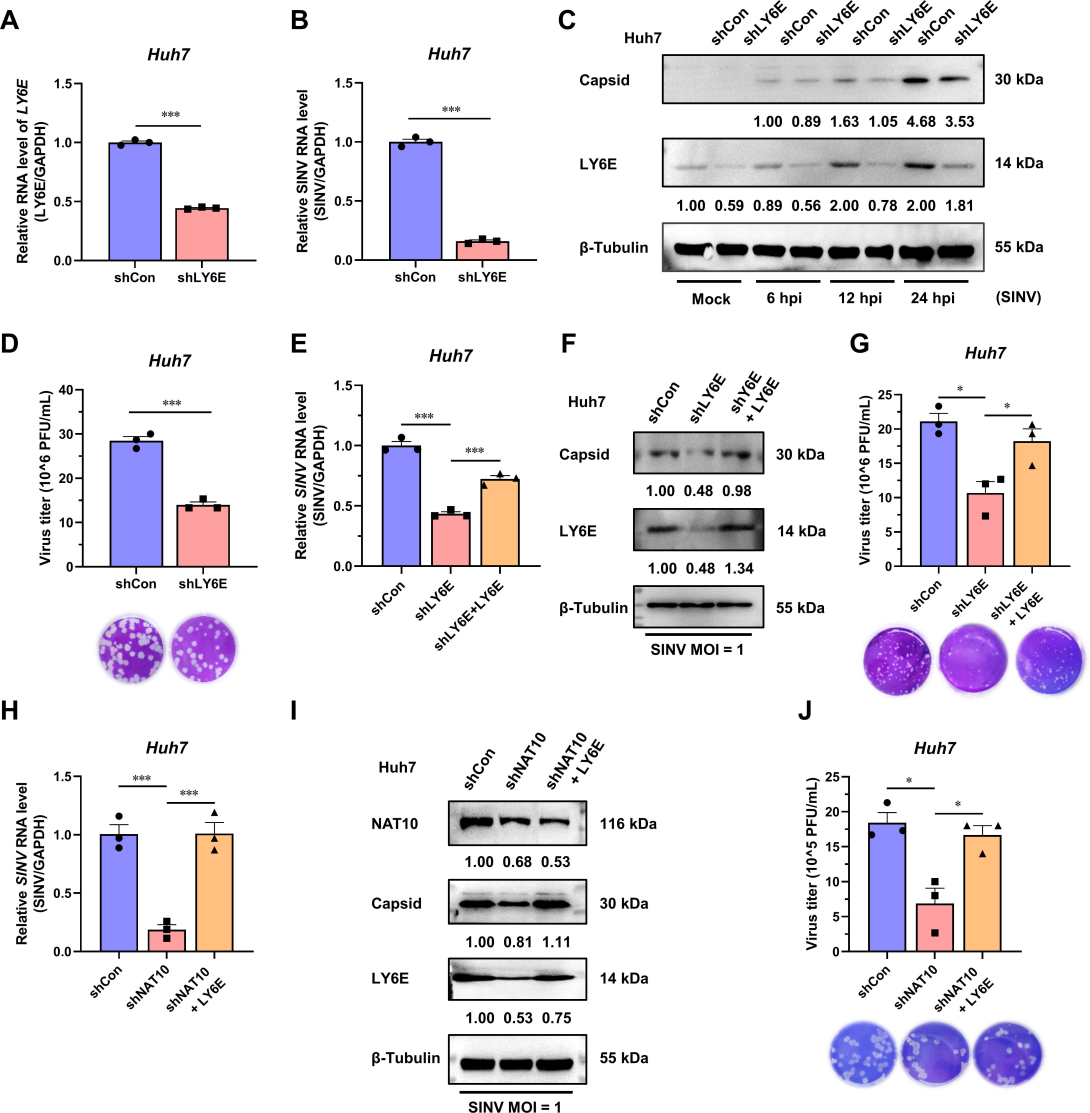
SINV is positively affected by NAT10 as it regulates the stability of the LY6E mRNA. (**A**) qRT-PCR analysis of *LY6E* mRNA expression in LY6E-KD Huh7 cells. (**B**) qRT-PCR analysis of the SINV RNA expression levels in LY6E-KD Huh7 cells at 24 hpi (MOI = 1). (**C**) Immunoblot analysis of the SINV capsid protein expression in LY6E-KD Huh7 cells at 6, 12, and 24 hpi (MOI = 1). (**D**) Plaque formation assay using the SINV infectious virions obtained from the LY6E-KD Huh7 cell culture medium at 24 hpi (MOI = 1). (**E**) qRT-PCR analysis of the SINV RNA expression levels in LY6E-KD Huh7 cells ectopically expressing LY6E and infected with SINV, 24 hpi (MOI = 1). (**F**) Immunoblot analysis of the SINV capsid protein abundance described in panel (**E**). (**G**) Plaque formation assay using the SINV infectious virions obtained from the culture supernatant described in panel (**E**). (**H**) qRT-PCR analysis of the SINV RNA expression levels in NAT10-KD Huh7 cells ectopically expressing LY6E and infected with SINV, 24 hpi (MOI = 1). (**I**) Immunoblot analysis of the SINV capsid protein abundance as described in panel (**H**). (**J**) Plaque formation assay for the SINV infectious virions obtained from the culture supernatant described in panel (**H**). Blots were quantified with ImageJ software and normalized to control levels. Data are presented as the means ± SEM (*n* = 3). ^*^P ≤ 0.05 and ^***^P ≤ 0.001 (A, B, and D, unpaired Student’s *t*-tests; E, G, H, and J, one-way ANOVA with Tukey’s multiple comparisons test).

As NAT10 regulates LY6E mRNA, the role of LY6E in SINV infection was investigated. The LY6E-KD Huh7 cells (Fig. 6A), wherein the levels of SINV RNA and capsid were significantly reduced, were generated (Fig. 6B and C). The virus titer in the culture supernatant was also reduced (Fig. 6D). We then exogenously expressed LY6E in LY6E-KD Huh7 cells, and the expression levels of the SINV RNA and capsid protein were restored (Fig. 6E and F), as well as the SINV titer in the culture supernatant (Fig. 6G). These results indicate that LY6E promotes the SINV infection.

As NAT10 depletion reduces SINV replication and the level of LY6E, it was suspected that the NAT10 may regulate SINV infection through *LY6E* mRNA modifications. To verify this hypothesis, the LY6E was ectopically expressed in NAT10-KD cells, rescuing the NAT10 depletion-driven SINV replication inhibition. The SINV RNA, capsid protein levels, and infectious virion titers in the supernatant were restored after LY6E supplementation (Fig. 6H through J). Collectively, these results demonstrate that NAT10 manipulates SINV infection by regulating *LY6E* mRNA stability via the ac4C modification within the 3′-UTR.

## DISCUSSION

The role of epitranscriptomic modifications in the viral life cycle has been extensively studied in different viruses, including HIV, EV71, influenza virus, flavivirus, coronavirus (CoV), and herpesviruses (17, 31–40). Specifically, methylation modifications, such as m6A, m1A, m5C, guanosine methylation, and 2-O’-methylation, have been discussed (41–45). Conversely, the role of acetylation in viral infections remains largely unexplored. In this study, we identified an important role for NAT10 in the regulation of the *alphavirus* infection via downstream *LY6E* mRNA stability.

The ac4C modification is present within the genomes of different viruses, including HIV-1 (4), EV71 (5), and influenza virus (46). Within these genomes, the ac4C modification has been implicated in regulating virus replication and pathogenesis. This included the silent mutagenesis of ac4C sites in HIV-1 that diminishes viral gag expression and the ac4C site mutation in the EV71 5′-UTR, which reduces pathogenicity in mice. Although a similar phenomenon might be present in the *alphavirus*, we failed to detect sufficient SINV genomic RNA reads when using acRIP-seq to generate the ac4C peak information required for alphavirus RNA analysis. Further analysis will thus be required to evaluate ac4C direct modifications in the *alphavirus* genome using high-resolution methods, such as cross-linking and immunoprecipitation high-throughput sequencing or nanopore direct RNA sequencing (47).

NAT10 primarily modifies different types of RNA in the nucleolus, localizes to the midbody, and regulates cytokinesis and microtubule acetylation (48). Normal tissue cells express NAT10 in the nucleus, whereas tumor cells translocate NAT10 to the cytoplasm, and this affects tumor formation. The localization of NAT10 in different subcellular compartments is influenced by its nuclear localization signals; the deletion of approximately 10 conserved residues in the C-terminus of the NAT10 affects nuclear localization, leading to cytoplasmic accumulation (49). For example, the deletion of the NAT10 nuclear localization signal promotes the migration and invasion of hepatocellular carcinoma cells (50). The EV71 infection promoted an increase in the protein expression and altered the subcellular localization of NAT10, indicating that the virus affects the host modification mechanisms to promote its replication (5). NAT10 is considered an mRNA regulator that may promote the translation and stability of mRNAs via ac4C modification (2, 3). For example, UNC-52-like kinase 1 (ULK1) is a target of NAT10 in neutrophils (51). Hence, the downregulation of NAT10 expression results in the decay of ULK1 transcripts and enhanced activation of the STING-IRF3 pathway, increasing the number of pyroptosis-inducing NLRP3 inflammasomes in neutrophils (52). In the current study, in SINV-infected NAT10-KD cells, one of the target genes, was highly related to IFN, i.e., *LY6E* (22). Although various other IFN-related genes, including calpain 2 (*CAPN2*) and arginosuccinate synthase 1 (*ASS1*) (53, 54), have been identified using NGS sequencing, the associated functional mechanisms have not yet been fully characterized.

LY6E is a 133-amino-acid glycosylphosphatidyl-inositol (GPI)-anchored cell-surface protein (55). The expression of LY6E is induced by type I IFN, and it is transcriptionally active in several tissues, including the liver, spleen, lungs, and brain (56). Aside from the identified primary functions, namely, the regulation of T cell activation, proliferation, development, tumor metastasis, and differentiation (55), LY6E is associated with viral infections. It may promote HIV-1 entry via enhanced virus-cell fusion (22, 57). Additionally, LY6E is a receptor for the mouse endogenous retroviral envelope syncytin-A (58).

Previous ISG screening studies have reported that LY6E enhances the infectivity of various genetically diverse viruses, including members of the *Flaviviridae* [yellow fever virus (YFV), dengue virus, and West Nile virus], *Togaviridae* [CHIKV, O’nyong nyong virus (ONNV)], *Retroviridae* (HIV-1), and *Orthomyxoviridae* (influenza A virus) families (59, 60). For instance, overexpressing LY6E enhances YFV replication in immortalized human *STAT1^−/^*^−^ fibroblasts (29). Meanwhile, ectopic LY6E expression in human lung malignant adenoma (A549), human hepatoma (Huh7.5), human embryonic kidney (HEK293T), or Syrian infant hamster kidney (BHK) cells does not enhance YFV replication. Furthermore, LY6E modulates HIV-1 infection in a CD4-dependent manner in target cells (22, 61). The mammalian innate immune response partially controls coronavirus infection through the action of IFNs (62, 63). IFNs inhibit viral infection by inducing several genes, including *LY6E* (64). LY6E effectively restricts cellular infections caused via several coronaviruses, including two severe acute respiratory syndrome CoVs (SARS-CoV and SARS-CoV-2) and Middle East respiratory syndrome CoV (MERS-CoV) (65, 66). LY6E restricts coronavirus entry into the cells by interfering with spike protein-mediated membrane fusion, which is largely dependent on its lipid raft-associated GPI anchor and the evolutionarily conserved L36 residue (67). In addition, the conservation of LY6E homologs in humans, rhesus monkeys, mice, bats, and camels demonstrates their strong protective role across species.

In the current study, LY6E depletion in Huh7 cells reduced SINV infection, while its ectopic expression restored SINV replication. A previous study reported that ONNV and SINV replication was downregulated in LY6E-knockout U2OS cells. Furthermore, lentivirus-induced LY6E overexpression did not promote SINV replication in STAT1^−/−^ fibroblasts (67). These findings suggest that the LY6E-induced promotion of viral replication might be cell-specific.

LY6E-enhanced viral infection involves diverse mechanisms. The enhancement of viral entry by LY6E depends on its GPI anchoring, which is rich in lipid raft microstructural domains that regulate membrane-dependent processes, such as endocytic transport and signaling (68). Additionally, LY6E modulates cytoskeleton re-arrangement (69) and regulates cell signaling in the host immune response. LY6E downregulates CD14, a key molecule in the TLR4/CD14/NF-κB pathway, inducing a negative feedback loop for innate immune activation and providing a new pathway for viral infection (70, 71). Nonetheless, the mechanism by which LY6E regulates *alphavirus* infection requires further investigation.

*Alphaviruses* are a transboundary group of viruses that infect insects, birds, and mammals (72, 73). As such, it is of interest to determine whether the invertebrate NAT10 counterparts function like that in other vertebrates. Dengue virus infection of Aag2 cells induces m6A modification in the transcriptome of *Aedes aegypti* (74), which lacks an IFN system. Hence, elucidating mosquito NAT10 functions in the *alphavirus* life cycle may clarify certain aspects, such as whether the evolutionary conserved N-acetyltransferase has different roles in different hosts.

**Fig 7:**
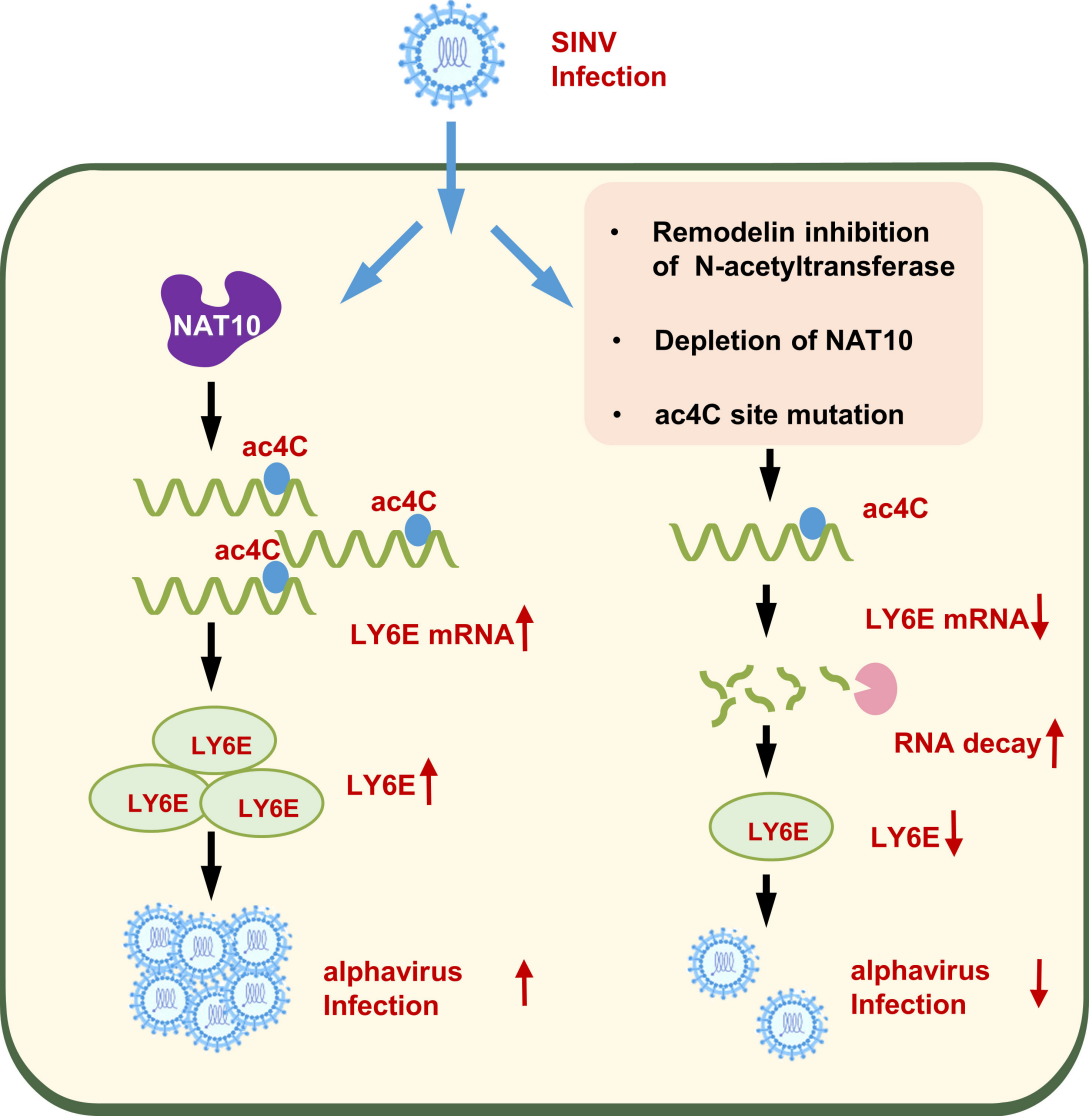
Working model showing how the loss of NAT10 reduces alphavirus replication. *Alphavirus* (SINV) infection upregulates NAT10 in host cells and promotes NAT10-mediated ac4C acetylation of *LY6E* mRNA transcripts, increasing LY6E expression and enhancing *alphavirus* replication.

In summary, the results demonstrate that ac4C plays an important role in *alphavirus* infection and that SINV regulates the ac4C modification system to facilitate efficient replication. Notably, NAT10 directly binds the *LY6E* mRNA and promotes its stability through ac4C modifications within the 3′-UTR region while promoting SINV replication (Fig. 7). However, certain limitations exist. First, As NAT10 loss enhanced degradation of the *LY6E* mRNA transcripts after viral infection, other genes may also contribute to the NAT10-mediated ac4C modifications that impact SINV replication. In addition, the ac4C modification regulates viral replication by affecting host mRNA; however, it remains unknown whether this modification also occurs in the *alphavirus* genome and what its regulatory role is. Nevertheless, this study revealed the essential functions of ac4C in understanding the *alphavirus* life cycle while providing a promising target for developing *alphavirus* antivirals.

## Data Availability

The RNA-seq data have been deposited in the NCBI GEO database under accession number GSE222477.
